# The International Veterinary Congress at Berne, Switzerland

**Published:** 1895-05

**Authors:** 


### SOCIETY PROCEEDINGS.

### SIXTH INTERNATIONAL CONGRESS OF VETERINARY MEDICINE AT BERNE, SWITZERLAND, 16TH TO 21ST SEPTEMBER, 1895.

THE JOURNAL is in receipt of an invitation addressed to the veterinary profession in America calling their attention to the Sixth International Congress of Veterinary Medicine, to be held at Berne, Switzerland, September 16th to 21st, 1895. They recall the circular issued on January 5, 1894, in which they announced:

1. That according to the decision of the Fifth International Congress, held at Paris in 1889, it was determined to hold the Sixth Congress at Berne.

2. The Committee of Organization has fixed the programme as given below. This is of the greatest interest in International Veterinary Sanitary Police, Public Hygeine, and Veterinary Medicine. Each of the subjects will be printed in French and German, and will be sent to the members of the Congress before the opening.

The members of the Congress will be all those veterinarians and persons interested who subscribe before the 15th of August next.

The subscription is fixed at ten francs ($2), which entitles the subscriber to all the publications of the Congress. Subscriptions should be addressed with postal order to the Treasurer, M. le Dr. Rabeli, Professor at the Veterinary School, Berne, Switzerland.

Programme of the Sixth International Congress of Veterinary Medicine :

*First Subject :* International veterinary sanitary police ; proposition for an international convention to regulate the commerce of cattle ; publication of an international bulletin concerning the contagious diseases of domestic animals.

Reporters : Berdez, of Berne ; Degive, of Brussels ; Hutzra, of Pesth, and Perroncito, of Turin.

*Second Subject :* Injections for diagnosis and immunity from the standpoint of veterinary sanitary police ; the results now obtained from them.

*a.* The value of mallein as a means of diagnosis of glanders.

Reporters : Beisswänger, of Stuttgart ; Nocard, of Alfort ; Preusse, of Danzig ; Schindelka, of Vienna.

*b.* The value of tuberculin as a means of diagnosis in tuberculosis.

Reporters : Baug, of Copenhagen ; Herr, of Berne ; Semmer, of St. Petersburg.

*c.* The pneumo-bacilline and its use in the diagnosis of pleuro-pneumonia.

Reporter : Arloing, of Lyons.

*d.* Preventive inoculation in symptomatic anthrax.

Reporters : Cornevin, of Lyons ; Herr, of Berne ; Strebel, of Fribourg ; Szpilmann, of Sembourg.

*e.* Inoculations for immunity and curative in tetanus in hog cholera and in pneumo-enteritis of the hog, etc.

Reporter : Schütz, of Berlin.

*Third Subject :* Tuberculous meat and public hygeine.

Reporters : Butel, of Meause ; Fleming, of England ; Guillebeau, of Bern, and Ostertag, of Berlin.

*Fourth Subject :* The influence of veterinary science on social development and on the augmentation of public health.

Reporter : Sydtin, of Karlsruhe.

*Fifth Subject :* Contagious pleuro-pneumonia ; exposé of the results in each country of the means employed to combat this epizootic disease.

Reporters : Ekkert, of St. Petersburg, for Russia ; Generali, of Modena, for Italy ; Hirzel, of Zurich, for Switzerland ; Leblanc, of Paris, for France and England and their colonies ; Liautard, of New York, for America ; Lindquist, of Stockholm, for Sweden-Norway ; Persu, of Bucharest, for Roumania, Turkey, and the Balkan States ; Rœcke, of Berlin, for Germany ; Thomassen, of Utrecht, for Holland, Belgium, Luxembourg, and Denmark ; Antero Viurrun, of Madrid, for Spain and Portugal.

General Reporter : M. Hirzel.

*Sixth Subject :* In accordance with a general expressed desire there will be a special section on anatomy—especially on a unification of anatomical nomenclature.

Reporters : Martin, of Zurick, and Rabeli, of Berne.

The reports of this Congress offer such an extended literature upon the most important subjects of veterinary medicine that it will be an opportunity for everyone to subscribe.

The manager of the JOURNAL OF COMPARATIVE MEDICINE will be glad to receive the subscriptions of American veterinarians and forward them. A list of American subscribers will be published in the Journals of June, July, August, and September. Address, inclosing $2,

DR. W. H. HOSKINS,
3452 Ludlow Street, Philadelphia, Pa.

